# Congenital absence of the anterior arch of the atlas: a normal variant

**DOI:** 10.1308/003588412X13373405384657

**Published:** 2012-10

**Authors:** D Thavarajah, P McKenna

**Affiliations:** Royal Berkshire Hospital, Reading,UK

**Keywords:** Congenital, Anomaly, Atlas, C1

## Abstract

Congenital absence of the anterior arch of the atlas is incredibly rare with only two cases reported previously in the literature. We present a third case of a medically fit patient who suffered neck trauma with an abnormal odontoid peg x-ray, which subsequently demonstrated a congenital non-fusion of the anterior vertebral arch of C1 on computed tomography. This case highlights the need to have an open diagnosis to include congenital anomaly when interpreting abnormal odontoid peg x-rays.

Congenital absence of the anterior arch of the atlas (C1) is incredibly rare with only two previously reported cases in the literature.^1^ In a study of congenital defects of the C1 arch in 1,153 postmortem dissections and cervical computed tomography(CT), there were no aplastic anterior arch anomalies seen.There were, however, 11 posterior arch defects identified, therefore a relatively more common occurrence of the atlas anomaly.^2^

## Case history

A 35-year-old healthy housewife presented to the emergency department with a 4-day history of neck pain. She injured her neck following a crash into a tyre wall safety barrier while go-karting. Her head jolted forward and hit the steering wheel. She was wearing a safety helmet. Over the following four days she felt no ease in the pain, which she described as a dull ache that was unremitting to analgesia.

**Figure 1:**
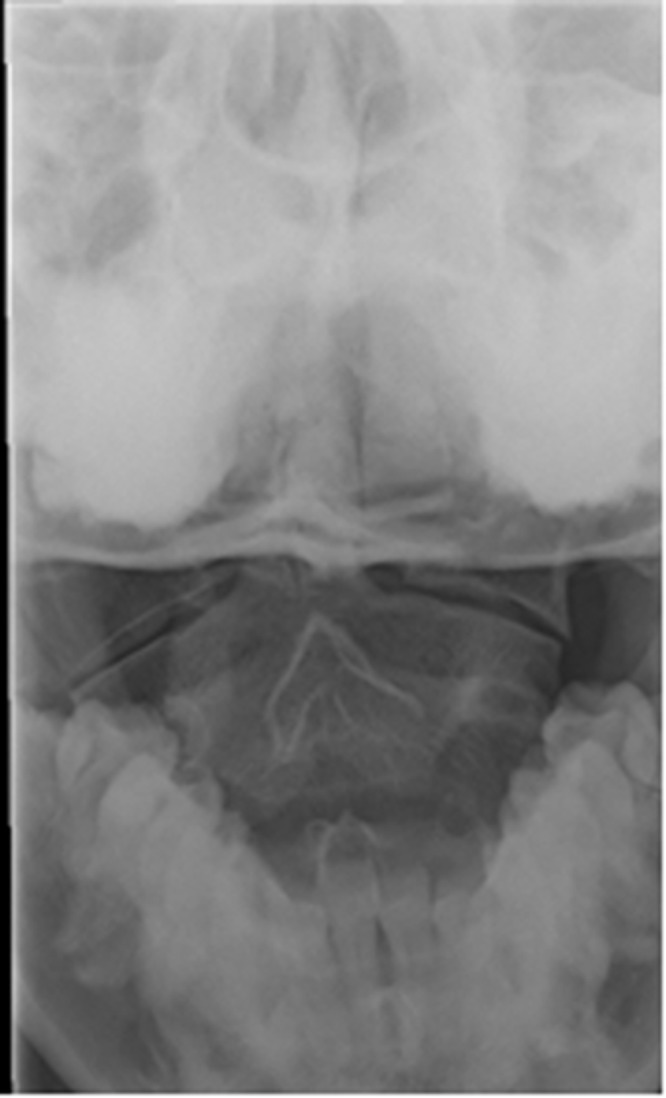
Odontoid peg view of asymmetrical lateral masses of the C1

**Figure 2:**
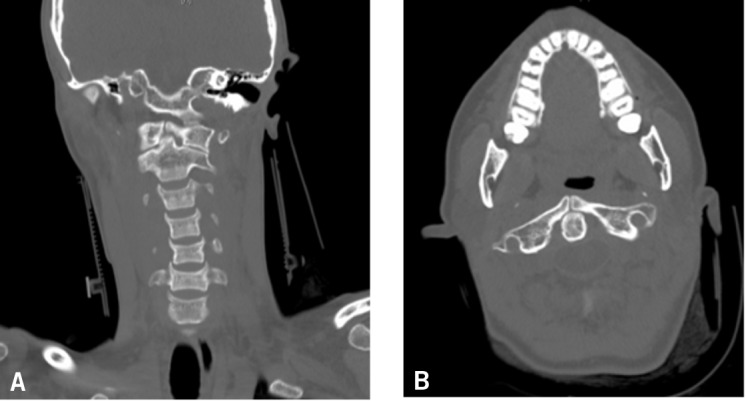
Coronal (A) and axial (B) computed tomography demonstrating non-fusion of the anterior arch of the atlas in the midline

On examination, she walked into the emergency department with a torticollis-type appearance to her head and neck. She had no neurological deficit. There was no cervical spine tenderness but there was left sternocleidomastoid tenderness. In view of the history of rapid deceleration and direct impact with the steering wheel, cervical spine views were obtained.The odontoid peg view gave cause for concern (Fig 1) and she was triple immobilised as a suspected atlas fracture. The lateral masses of the atlas appeared asymmetrical. CT demonstrated the rare congenital anomaly: a congenital absence and non-fusion of the anterior arch of C1 (Fig 2). The patient was mobilised and discharged home with analgesia for a whiplash injury with no sequelae.

## Discussion

The cause of a congenital absence of the arch of the atlas is due to failure of fusion of the synchondroses.It is during the seventh week of gestation that the ossification of the atlas begins, initially with ossification of the lateral masses (20% of newborns have an ossification centre in the anterior arch) that then extends dorsally. By the age of four the neural arches have fused posteriorly.^3,4^

At birth, the anterior arch of the atlas is commonly cartilaginous and forms ventral extensions from the lateral masses with fusion occurring by the ages of six to eight years.^3,5^ The lines of union extend across the anterior portions of the superior articular facets.

Attachments to the anterior arch include the longus colli muscle and the anterior longitudinal ligament to the anterior turbercle, which is present in the centre anteriorly. Posterior to the anterior arch is the articular surface with the dens, which has articular cartilage.

The upper and lower borders of the anterior arch give attachment to the anterior atlanto-occipital membrane and the anterior atlanto-axial ligament respectively.The former connects it with the occipital bone above and the latter with the axis below.

## Conclusions

The clinical significance of this anomaly despite an abnormal odontoid peg view, which raised the suspicion of fracture, is that congenital anomaly should be borne in mind when assessing the patient and x-rays. This patient had not previously complained of neck pain or had any neurological compromise so it was felt that she had a stable atlas and did not warrant any further intervention.

**Figure 3:**
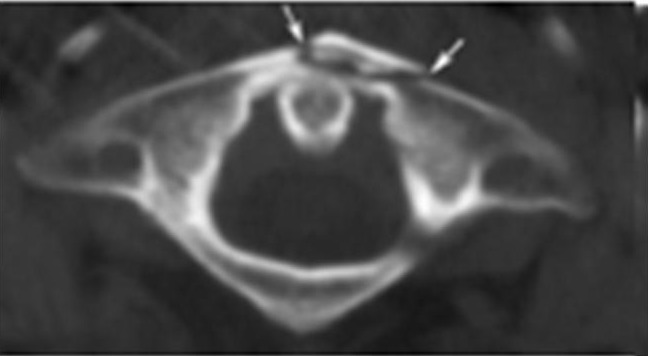
Axial computed tomography demonstrating a traumatic anterior arch atlas fracture

Comparison can be made to the rare traumatic anterior atlas arch fracture, also referred to as a ‘plough’ fracture, whereby in a hyperextension-type injury forward propulsion of the odontoid peg shears off the anterior arch of the atlas in a plough-type fashion (Fig 3).^6^
